# Characteristics and outcome of COVID-19 patients admitted to the ICU: a nationwide cohort study on the comparison between the first and the consecutive upsurges of the second wave of the COVID-19 pandemic in the Netherlands

**DOI:** 10.1186/s13613-021-00978-3

**Published:** 2022-01-13

**Authors:** Dave A. Dongelmans, Fabian Termorshuizen, Sylvia Brinkman, Ferishta Bakhshi-Raiez, M. Sesmu Arbous, Dylan W. de Lange, Bas C. T. van Bussel, Nicolette F. de Keizer, the Dutch COVID-19 Research Consortium, Dirk P Verbiest, Leo F te Velde, Erik M van Driel, Tom Rijpstra, Paul W. G. Elbers, Lyuba Georgieva, Eva Verweij, Remko M de Jong, Freya M van Iersel, Dick T. J. J. Koning, Els Rengers, Nuray Kusadasi, Michiel L Erkamp, Roy van den Berg, Cretièn J. M. G. Jacobs, Jelle L Epker, Annemiek A Rijkeboer, Martha T de Bruin, Peter Spronk, Annelies Draisma, Dirk Jan Versluis, Lettie A. E. van den Berg, Marissa Vrolijk-de Mos, Judith A Lens, D Jannet Mehagnoul-Schipper, Diederik Gommers, Johan G Lutisan, Martijn Hoeksema, Ralph V Pruijsten, Hans Kieft, Jan Rozendaal, Fleur Nooteboom, Dirk P Boer, Inge T. A. Janssen, Laura van Gulik, M Peter Koetsier, Vera M Silderhuis, Ronny M Schnabel, Ioana Drogt, Wouter de Ruijter, Rob J Bosman, Tim Frenzel, Louise C Urlings-Strop, Allard Dijkhuizen, Ilanit Z Hené, Arthur R de Meijer, Jessica W. M. Holtkamp, Nynke Postma, Alexander J. G. H. Bindels, Ronald M. J. Wesselink, Eline R van Slobbe-Bijlsma, Peter H. J. van der Voort, Bob J. W. Eikemans, Michel G. W. Barnas, Barbara Festen-Spanjer, Maarten van Lieshout, Niels C Gritters, Martijn van Tellingen, Gert B Brunnekreef, Joyce Vandeputte, Tom P. J. Dormans, Marga E Hoogendoorn, Mart de Graaff, David Moolenaar, Auke C Reidinga, Jan Jaap Spijkstra, Ruud de Waal

**Affiliations:** 1 National Intensive Care Evaluation (NICE) Foundation, PO Box 23640, 1100 EC Amsterdam, The Netherlands; 2 Department of Intensive Care Medicine, Amsterdam UMC, University of Amsterdam, Meibergdreef 9, 1105 AZ Amsterdam, The Netherlands; 3 Department of Medical Informatics, Amsterdam Public Health Research Institute, Amsterdam UMC, University of Amsterdam, Meibergdreef 9, 1105 AZ Amsterdam, The Netherlands; 4 Department of Intensive Care Medicine, Leiden University Medical Center, Albinusdreef 2, 2333 ZA Leiden, The Netherlands; 5 Department of Intensive Care Medicine, University Medical Center, University of Utrecht, Heidelberglaan 100, 3584 CX Utrecht, The Netherlands; 6 Department of Intensive Care Medicine, Maastricht University Medical Centre+, P. Debyelaan 25, 6229 HX Maastricht, The Netherlands; 7 Maastricht University, Care and Public Health Research Institute (CAPHRI), Universiteitssingel 40, 6229 ER Maastricht, The Netherlands

**Keywords:** COVID-19, Coronavirus, Mortality, Outcome, Intensive Care, Critical Care

## Abstract

**Background:**

To assess trends in the quality of care for COVID-19 patients at the ICU over the course of time in the Netherlands.

**Methods:**

Data from the National Intensive Care Evaluation (NICE)-registry of all COVID-19 patients admitted to an ICU in the Netherlands were used. Patient characteristics and indicators of quality of care during the first two upsurges (N = 4215: October 5, 2020–January 31, 2021) and the final upsurge of the second wave, called the ‘third wave’ (N = 4602: February 1, 2021–June 30, 2021) were compared with those during the first wave (N = 2733, February–May 24, 2020).

**Results:**

During the second and third wave, there were less patients treated with mechanical ventilation (58.1 and 58.2%) and vasoactive drugs (48.0 and 44.7%) compared to the first wave (79.1% and 67.2%, respectively). The occupancy rates as fraction of occupancy in 2019 (1.68 and 1.55 vs. 1.83), the numbers of ICU relocations (23.8 and 27.6 vs. 32.3%) and the mean length of stay at the ICU (HRs of ICU discharge = 1.26 and 1.42) were lower during the second and third wave. No difference in adjusted hospital mortality between the second wave and the first wave was found, whereas the mortality during the third wave was considerably lower (OR = 0.80, 95% CI [0.71–0.90]).

**Conclusions:**

These data show favorable shifts in the treatment of COVID-19 patients at the ICU over time. The adjusted mortality decreased in the third wave. The high ICU occupancy rate early in the pandemic does probably not explain the high mortality associated with COVID-19.

**Supplementary Information:**

The online version contains supplementary material available at 10.1186/s13613-021-00978-3.

## Introduction

Various studies reported high mortality rates among COVID-19 patients admitted to the Intensive Care Unit (ICU). These rates appear to be higher than reported among ICU patients with other types of viral pneumonia [1]. During the COVID-19 pandemic in the Netherlands, many ICUs were scaled up above their maximum capacity. Of note, the Netherlands healthcare system has less ICU beds per capita than other European countries, i.e. 6.4 per 100,000. For instance, Belgium with 15.9 or Germany with 29.2 have significantly more ICU beds per 100,000 inhabitants [2]. This combined with both the high number of patients and their longer length of stay (when compared to average ICU patients) caused a limitation in the numbers of available ICU beds. This potentially has had adverse effects on the quality of care, and may have increased the mortality risk. To utilize ICU beds most effectively, preventing overload due to new cases of high emergency (related or not related to COVID-19), ICU patients were transferred between regional and (inter)national ICUs [3]. A relocation is not risk free, especially for mechanically ventilated patients [4].

In the present study, we describe the clinical characteristics, the length of ICU stay, relocation rate, and mortality of ICU patients infected with SARS-CoV-2 during the first wave, the period in-between and the consecutive upsurges thereafter of the COVID-19 pandemic in the Netherlands. The aim is to examine whether there is evidence for an improvement in the quality of care for COVID-19 patients at the ICU, as indicated by a shorter duration of stay, a lower relocation rate, and a lower hospital mortality rate during the epidemic upsurges after the first wave. This will also be helpful to understand whether the high mortality risk associated with COVID-19 is an inevitable effect of the virus infection itself or was also due to the (imminent) inadequate response of the health care system in the early phases of the pandemic.

## Methods

### Data

The National Intensive Care Evaluation (NICE) registry is a voluntary quality registry in which all Dutch ICUs participate. This registry includes prospectively collected clinical data of all patients admitted to an ICU and includes demographics, major comorbidities, physiology, clinical course, ICU length of stay, in-hospital mortality and mechanical ventilation. Of note, mechanical ventilation in our data set includes invasive as well as non-invasive ventilation, there is no differentiation between the two. High flow nasal oxygen (HFNOT) is excluded as a mechanical ventilation mode. These routinely collected data are extracted from the electronic health record and after validation uploaded on a monthly basis. According to clear definitions and using rigorous data quality checks maximum data quality was ensured [5]. The purpose of NICE is to provide feedback on performance indicators to ICUs, thus enabling ICUs to monitor and improve their quality of care. From the start of the COVID-19 outbreak in the Netherlands, the Dutch government requested all ICUs to record all suspected and confirmed COVID-19 patients admitted to the ICU. Therefore, the existing NICE data infrastructure was expanded with a module allowing for daily recording of admission- and discharge dates, and survival status at ICU- and hospital discharge of COVID-19 patients. Clinical data of the COVID-19 patients were linked afterwards when uploaded according to the regular NICE processes. NICE data infrastructure allowed that COVID-19 patients could be accurately tracked throughout subsequent hospital admissions. This made it possible to combine the length of ICU stay between hospitals and final hospital survival status, thus taking transfers into account.

A confirmed COVID-19 patient was defined as follows: a positive SARS-CoV-2 Reverse transcription polymerase chain reaction (RT-PCR) on a nasopharyngeal swab or a CT-scan consistent with COVID-19 (i.e., a CO-RADS score of ≥ 4 in combination with the clinical diagnosis viral pneumonia) [6].

**Fig. 1:**
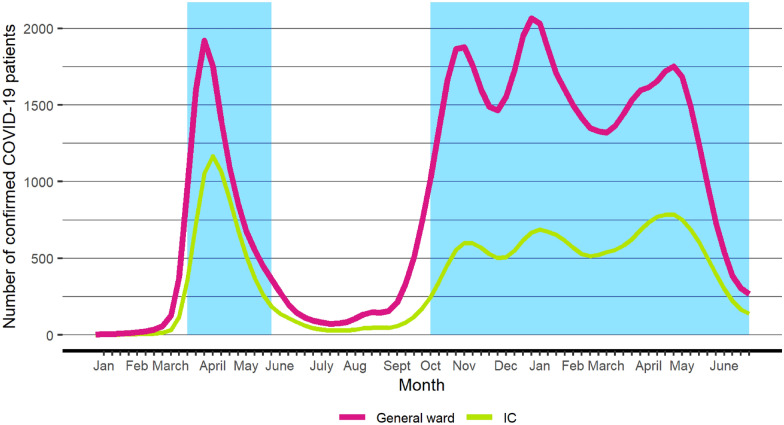
Number of COVID-19 patients present at the general ward in hospital and at the ICU during the pandemic in the Netherlands

In 2020 and 2021, two big waves of ICU admissions due to COVID-19 infections were observed in the Netherlands with a distinct period in between (Fig. 1 and Additional file 1: Fig. S1). We decided to categorize calendar time into the following time periods under investigation: the first wave (February 1–May 24, 2020), the episode between the waves (May 25–October 4, 2020), the second wave—first and second upsurge (October 5, 2020–January 31, 2021), and the second wave—final upsurge (February 1, 2021–June 30, 2021). Albeit the final upsurge of the second wave does not appear to be a separated wave, that is, did not follow after a substantial decline in cases towards zero, for ease of reference we call the final upsurge of the second wave the ‘third wave’. The period of this third wave is chosen based on visual inspection and because of the fact that the vaccination program in the Netherlands commenced on January 6th 2021 with a focus on vulnerable and older people at first. About half a million people had received their first vaccination on February the first. From then on vaccination was sped up and about a million people received a vaccination per month. Patients categorized in a time period under investigation were analyzed until death or hospital discharge, that is, beyond the time period’s limits. Thus, a death event in period two of a patient admitted in period one was assigned to period one.

The Medical Ethics Review Committee of the Academic Medical Center waived the need for informed consent [reference number W21_091 # 21.102].

### Statistical analyses

The number of patients, patient- and treatment characteristics, duration of total ICU- and hospital stay (combining subsequent hospital and ICU stays within a treatment trajectory of an unique patient), occupancy rate calculated as the number of occupied beds at the day of ICU admission of the patient at the pertinent ICU as percentage of the daily average in 2019 specific for that ICU, and relocation rate were described for the four periods separately. The in-hospital mortality, that is death at the ICU or death at the hospital ward after ICU discharge, was estimated as percentage and analyzed in a logistic regression model with wave (first, period in-between, second and third) as the main covariate. The Odds ratios (ORs) of hospital mortality were adjusted for age, sex, BMI, the APACHE-IV mortality probability, and the ICU occupancy rate at the day of ICU admission. The length of ICU stay was analyzed in a multivariable Cox regression model with ICU discharge as outcome event. Again, wave was the main variable of interest and the Hazard ratio (HR) of ICU discharge was adjusted for the above-mentioned co-variates.

A higher or lower mortality rate may lead to a shorter or longer mean length of ICU stay, respectively. To examine whether shifts in ICU length of stay are independent of possible shifts in mortality, the analysis was performed in two different ways: first with time of death included as an ICU discharge event, and second with time of death included as reason for censoring. The last analysis is probably more valid, as it reduces the influence of short stays caused by early death events, and thus reflects trends in length of stay assuming that all patients survived the ICU admission. If no substantial differences between both methods are found, this indicates that shifts in mortality did not influence shifts in mean length of stay.

All analyses were performed using the R statistical environment (version 3.6.1) (R Foundation for Statistical Computing, Vienna, Austria). We report P values and effect estimates with 95% confidence intervals.

## Results

### Patient and treatment characteristics

From February 2020 until the first of July 2021 there were 12,723 COVID-19 patients admitted to 78 Dutch ICUs. Of these, we excluded 5.4%, since their clinical data were not available, leaving 12,030 patients (94.6%) for the final analyses. Of note 2.6% of the patients were not discharged from the hospital at the time of the final analysis.

In Table 1, the demographic and clinical characteristics of included patients are given, for the full cohort, and stratified by period (i.e. Wave 1, Period in-between, Wave 2, and Wave 3). During Wave 1 compared to Wave 2, the proportion of patients aged 75 years or older was 13.6 vs. 17.0% (Additional file 1: Table S1). In Wave 3 the proportion of this group decreased to 10.7%. The mean age increased almost a year from Wave 1 to Wave 2 (63.1–64.3) but in Wave 3 the mean age decreased 3 years (61.3). In accordance with this finding, the proportion of patients with 2 or more comorbidities was higher during Wave 2 compared to Wave 1 (12.9% vs. 7.5%) and decreased in Wave 3 (9.0%). The prevalence figures for each of the comorbidities were higher during Wave 2, compared to either Wave 1 or the period in-between, whereas in Wave 3 these decreased again (Additional file 1: Table S1). In particular, renal insufficiently increased from 2.7% in Wave 1 to 5.9% in Wave 2 and decreased to 3.4 in Wave 3. The APACHE-IV estimated risk of in-hospital mortality remained relatively stable with a slight increase in Wave 2 (Table 1). Over time there was an increase in the percentage of obese patients BMI (> 30 kg/m2).

**Table 1:** Patient characteristics

Characteristic	Total	Wave 1	In-between	Wave 2	Wave 3	P value Wave 2 vs. Wave 1	P value Wave 3 vs. Wave 1
Number All	12,723	2812	505	4471	4935		
Number with linkage clinical records, N (%)	12,030 (94.6)	2733 (97.2)	480 (95.0)	4215 (94.3)	4602 (93.3)		
Age, Mean (SD)	62.8 (11.6)	63.1 (11.3)	61.8 (12.7)	64.3 (11.3)	61.3 (11.7)	< 0.001	< 0.001
Gender, N (%) Male	8389 (69.7)	1973 (72.2)	325 (67.7)	3013 (71.5)	3078 (66.9)	0.5392	< 0.001
BMI, N (%) < 18.5 kg/m2	49 (0.4)	9 (0.3)	5 (1)	18 (0.4)	17 (0.4)	< 0.001	< 0.001
18.5–25 kg/m2	2303 (19.1)	597 (21.8)	93 (19.4)	815 (19.3)	798 (17.3)		
25–30 kg/m2	4811 (40)	1218 (44.6)	188 (39.2)	1663 (39.5)	1742 (37.9)		
30–35 kg/m2	2892 (24)	568 (20.8)	104 (21.7)	1014 (24.1)	1206 (26.2)		
35–40 kg/m2	1157 (9.6)	185 (6.8)	55 (11.5)	437 (10.4)	480 (10.4)		
> 40 kg/m2	558 (4.6)	88 (3.2)	20 (4.2)	167 (4)	283 (6.1)		
Unknown	260 (2.2)	68 (2.5)	15 (3.1)	101 (2.4)	76 (1.7)		
BMI, Mean (SD)	29.5 (5.5)	28.7 (4.9)	29.4 (5.6)	29.4 (5.4)	30.0 (5.8)	< 0.001	< 0.001
Number of chronic comorbidities*, N (%) 0	7105 (59.1)	1760 (64.4)	267 (55.6)	2261 (53.6)	2817 (61.2)	< 0.001	< 0.001
1	3691 (30.7)	769 (28.1)	168 (35)	1387 (32.9)	1367 (29.7)		
2	1016 (8.4)	174 (6.4)	34 (7.1)	452 (10.7)	356 (7.7)		
> 2	218 (1.8)	30 (1.1)	11 (2.3)	115 (2.7)	62 (1.3)		
APACHE-IV mortality probability Mean (SD)	0.26 (0.18)	0.26 (0.18)	0.25 (0.20)	0.28 (0.18)	0.25 (0.16)	< 0.001	0.0236
Diabetes at ICU admission N (%)	2726 (22.7)	521 (19.1)	142 (29.6)	1091 (25.9)	972 (21.1)	< 0.001	0.0369
Acute renal failure in first < 24 h of ICU admission N (%)	850 (7.1)	252 (9.2)	34 (7.1)	289 (6.9)	275 (6)	< 0.001	< 0.001
PaO2 at ICU admission (in mmHg) Mean (SD)	78.0 (31.6)	85.0 (35.9)	78.0 (27.6)	76.2 (29.8)	75.6 (30.3)	< 0.001	< 0.001

^*^Included comorbidities: Immunological, renal, respiratory, and cardiovascular insufficiency, cirrhosis, and malignancy

Wave 1, February, 2020–May 24, 2020

Period in-between, May 25, 2020–October 4, 2020

Wave 2: first and second upsurge of Wave 2, October 5, 2020–January 31, 2021

Wave 3: final upsurge of Wave 2, February 1–June 30, 2021

As presented in Table 2, the mean (sd) time spend in hospital before admission to the ICU increased almost a day, from 1.7 (3.0) days in the Wave 1 to 2.4 (9.6) days in the Wave 3. The percentage of patients who were mechanically ventilated at the moment of admission decreased between Wave 1 from 48.1% to 25.1% in Wave 2 and this remained in the same range in Wave 3 (24.7%). The percentage of patients who were mechanically ventilated in the first 24 h of admission was 79.1% vs. 58.1% for Wave 1 and Wave 2, respectively, and this remained stable in Wave 3 (58.2%). The use of vasoactive drugs decreased as well (67.2% vs. 48.0%) and in Wave 3 this was even lower (44.7%). The ICU occupancy rates were on average lower both during Wave 3 and Wave 2 and the period in-between than the occupancy rate during Wave 1 (168%, 155% and 109% vs. 183%, respectively). In accordance with this finding, the relocation rates during the last periods were lower than the relocation rate during Wave 1 (23.8%, 27.6%, and 22.7% vs. 32.3%, respectively).

**Table 2:** Treatment characteristics and crude outcome

Characteristic	Total	Wave 1	In-between	Wave 2	Wave 3	P value Wave 2 vs. Wave 1	P value Wave 3 vs. Wave 1
Mechanical ventilation at ICU admission, N (%)	3607 (30.0)	1315 (48.1)	98 (20.4)	1057 (25.1)	1137 (24.7)	< 0.001	< 0.001
Mechanical ventilation in first 24 h of ICU admission, N (%)	7537 (62.7)	2161 (79.1)	246 (51.2)	2450 (58.1)	2680 (58.2)	< 0.001	< 0.001
Vasoactive drugs in first 24 h of ICU admission, N (%)	6129 (50.9)	1837 (67.2)	209 (43.5)	2024 (48.0)	2059 (44.7)	< 0.001	< 0.001
ICU occupancy rate (fraction of occupancy in 2019) at admission (in quintiles), N (%) 0.11–1.15	2052 (17.1)	413 (15.1)	322 (67.1)	791 (18.8)	526 (11.4)	< 0.001	< 0.001
1.15–1.39	2437 (20.3)	298 (10.9)	79 (16.5)	1043 (24.7)	1017 (22.1)		
1.39–1.66	2481 (20.6)	387 (14.2)	33 (6.9)	967 (22.9)	1094 (23.8)		
1.66–2.07	2486 (20.7)	670 (24.5)	16 (3.3)	786 (18.6)	1014 (22.0)		
2.07–5.49	2500 (20.8)	959 (35.1)	28 (5.8)	573 (13.6)	940 (20.4)		
Unknown	74 (0.6)	6 (0.2)	2 (0.4)	55 (1.3)	11 (0.2)		
ICU occupancy rate (fraction of occupancy in 2019) at admission Mean (SD)	1.64 (0.55)	1.83 (0.62)	1.09 (0.42)	1.55 (0.47)	1.68 (0.52)	< 0.001	< 0.001
Stay at the hospital pre-ICU (days) Mean (SD)	2.2 (9.6)	1.7 (3.0)	1.0 (18.6)	2.5 (10.9)	2.4 (9.6)	< 0.001	< 0.001
Transfer to other ICU, N (%)	3249 (27.0)	884 (32.3)	109 (22.7)	1163 (27.6)	1093 (23.8)	< 0.001	< 0.001
Length of ICU stay, days Mean (SD)	17.5 (17.6)	20.7 (20.6)	16.3 (16.1)	17.2 (16.9)	16.0 (16.3)	< 0.001	< 0.001
Length of hospital stay, days Mean (SD)	31.7 (55.1)	37.8 (78.3)	34.6 (70.3)	31.4 (51.5)	28.1 (36.3)	< 0.001	< 0.001
ICU mortality, N (%)	2916 (24.2)	735 (26.9)	109 (22.7)	1161 (27.5)	911 (19.8)	0.5704	< 0.001
In-hospital mortality, N (%)	3377 (28.1)	818 (29.9)	130 (27.1)	1350 (32.0)	1079 (23.4)	0.0692	< 0.001

Wave 1, February, 2020–May 24, 2020

Period in-between, May 25, 2020–October 4, 2020

Wave 2: first and second upsurge of Wave 2, October 5, 2020–January 31, 2021

Wave 3: final upsurge of Wave 2, February 1–June 30, 2021

### Outcome: in-hospital mortality

**Fig. 2:**
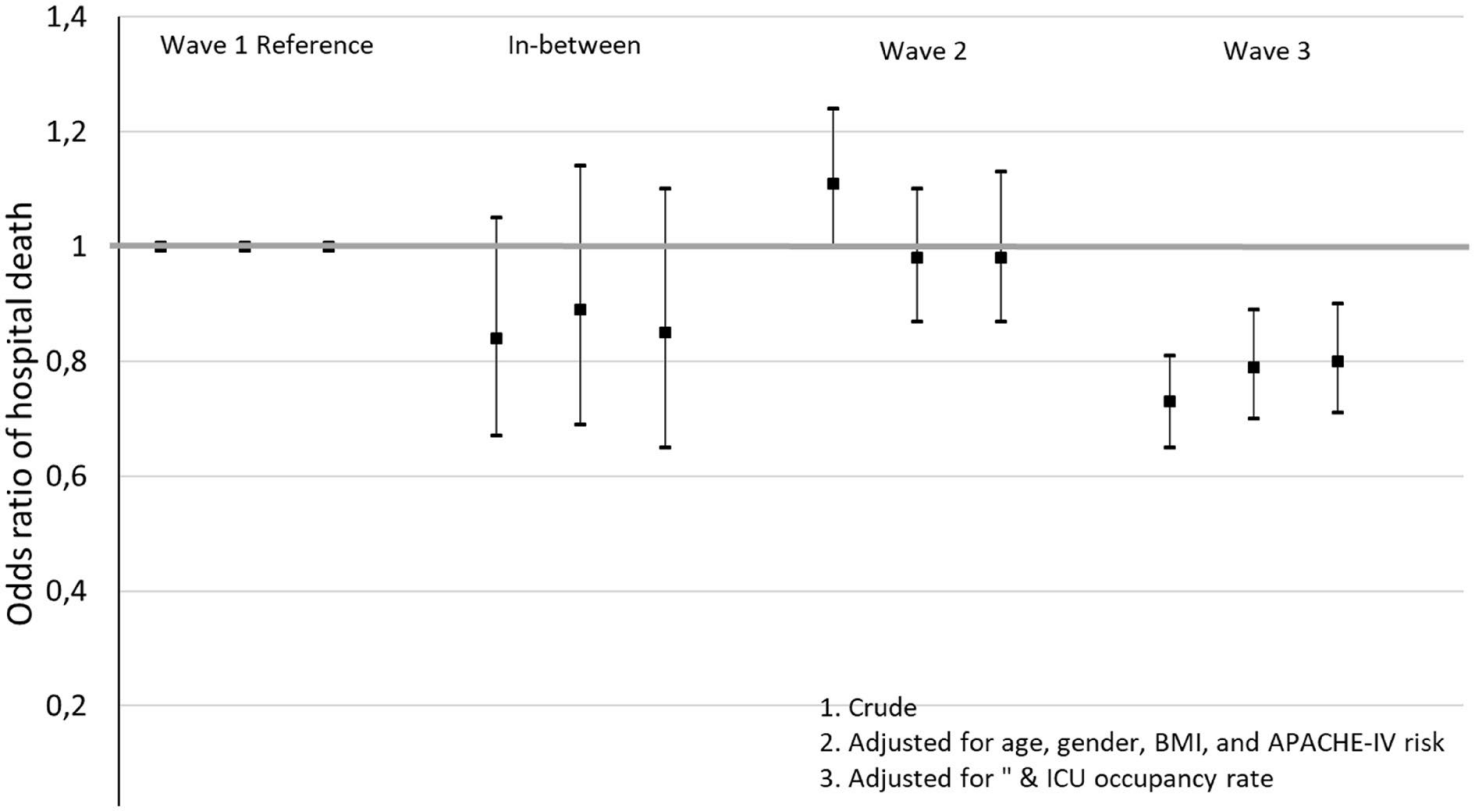
Crude and adjusted logistic regression showing Odds ratios of hospital death during Wave 2 and Wave 3, and the period in-between compared to Wave 1 (see Additional file 1: Table S2). Wave 1, February, 2020–May 24, 2020. Period in-between, May 25, 2020–October 4, 2020. Wave 2: first and second upsurge of Wave 2, October 5, 2020–January 31, 2021. Wave 3: final upsurge of Wave 2, February 1–June 30, 2021

The crude percentage of patients who died in the hospital during Wave 1 was 29.9%. This crude hospital mortality decreased to 27.1% in the in-between period, increased to 32.0% during Wave 2 and then decreased to 23.4% in Wave 3 (Table 2). After adjustment for age, gender, BMI, and the APACHE–IV mortality probability, the odds of hospital death in Wave 3 was significantly lower than the odds of hospital death in Wave 1: OR = 0.79 [0.70–0.89]. Further adjustment for the ICU occupancy rate at the day of ICU admission did not modify these results: OR = 0.80 [0.71–0.90] (Fig. 2, Additional file 1: Table S2).

### Outcome: length of ICU stay

**Fig. 3:**
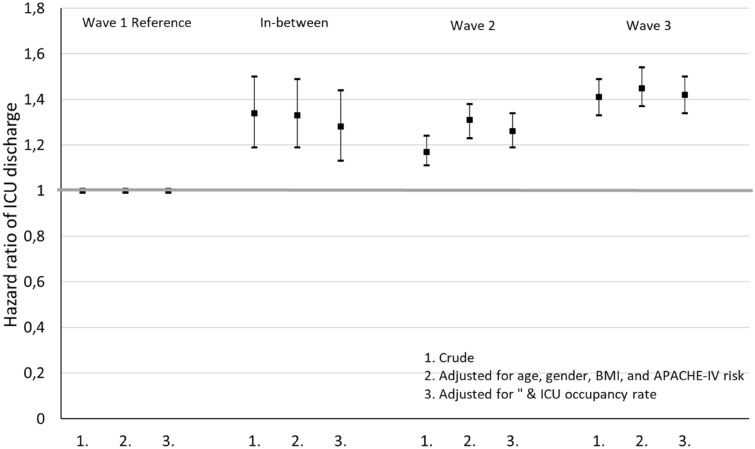
Crude and adjusted Cox regression showing Hazard ratios of ICU discharge during Wave 2 and Wave 3, and the period in-between compared to Wave 1 (see Additional file 1: Table S3, death as censoring event). A Hazard ratio of ICU discharge higher than 1.00 implies a comparatively high rate of discharge and, thus, a shorter length of stay at the ICU. Wave 1, February, 2020–May 24, 2020. Period in-between, May 25, 2020–October 4, 2020. Wave 2: first and second upsurge of Wave 2, October 5, 2020–January 31, 2021. Wave 3: final upsurge of Wave 2, February 1–June 30, 2021

The average length of ICU stay was 20.6 days during Wave 1 and this decreased to 16.3 days during the in-between period and to 17.2 days during Wave 2 and decreased even further to 16.0 days in Wave 3 (Table 2). The significantly shorter duration until ICU discharge during Wave 2 and Wave 3 compared to Wave 1 was also shown in a Cox regression model with death as censoring event and after adjustment for age, gender, BMI, and the APACHE–IV mortality probability (HR = 1.31 [1.23–1.38] and 1.45 [1.37–1.54]) (Fig. 3, Additional file 1: Table S3). Further adjustment for the ICU occupancy rate did not substantially modify this result. Similar results were found when death was included as discharge event instead of censoring event (Additional file 1: Table S3).

## Discussion

In this study, we compared the second wave, split into two parts, with the first wave of ICU COVID-19 admissions in the Netherlands. We found a shift to patients with a less favorable prognosis at ICU admission during the first and second upsurge of Wave 2, i.e. towards older patients with more comorbidities and a higher APACHE-IV mortality probability. In addition, we found a shift to favorable outcome and treatment characteristics, that is a shorter mean length of stay at the ICU, less relocations to other hospitals, and a lower number of patients treated with mechanical ventilation and vasopressor drugs. Furthermore, during the final upsurge of Wave 2 (‘Wave 3’) the age declined. It is unfortunate that no improvement in the patient survival could be established up until Wave 3. However, we cannot exclude that selection of patients with the best chance of ICU survival in Wave 1, due to health care driven scarce in ICU beds in the Netherlands, might have led to underestimation of the differences in survival.

We found that case-mix adjusted hospital mortality of ICU patients decreased in Wave 3 of the pandemic in the Netherlands. This suggests that insights in the pathophysiology of COVID-19 with increasingly appropriate treatments and vaccination and improved national healthcare system response by effective logistic- and organizational preparations (such as patient spreading) entailed more efficient care for COVID-19 patients.

Comparing our cohort of COVID-19 patients with respect to age to other cohorts of ICU patients shows that our patients are comparable to patients in other countries described in a review by Serafin et al. [7]. An important observation is that the age of COVID-19 patients in our study was higher during Wave 2. This was also observed in a study comparing the first and second wave in France [8]. In a large cohort of ICU patients in Brazil pre-vaccination, the age of patients admitted to the ICU declined over time [9]. In our view, the age of patients admitted to the ICU is influenced by selection of patients on one hand and the vaccination program on the other. For instance, in the Netherlands general practitioners played an important role in making it possible for patients to stay at home and receive tailored (palliative) care [10]. Scarcity of resources, i.e. availability of ICU beds, could also influence patient selection. The ICU occupancy rate was, as we showed, on average much higher during Wave 1 than during the in-between period and Wave 2 and Wave 3 (Table 2). Whether or not this truly has played a role in the selection of patients remains an important question. During Wave 3 the vaccination program started targeting vulnerable and older subjects at first.

In our cohort the number of comorbidities per patient was higher in Wave 2 and declined in Wave 3. In the cohort described by ICNARC from the UK there was also a slight increase in comorbidities [11], in contrast with the French cohort, where there was a non-significant decrease of comorbidities. These trends in number of comorbidities could again be explained by changing selection criteria over time. In our cohort we feel that a more liberal attitude towards admission of patients with comorbidities was the case in Wave 2. The decline in comorbidities in Wave 3 may be associated with the decrease in age due to the vaccination program. Moreover, the differences between countries could also be explained by the number of available ICU beds per country [2].

With respect to the need for mechanical ventilation, our study showed a decrease between Wave 1 and Waves 2 and 3. The same pattern was observed in Germany, where the percentage of ICU patients on mechanical ventilation decreased from 64 to 54% [12]. In France the need for invasive ventilation (this does thus not include non-invasive mechanical ventilation) in ICU dropped from 88 to 64% [8]. The reason for this decrease in our cohort is clearly not a decline in severity of illness. It could, however, be due to the fact that alternative means of providing oxygen support were used more often in ICU patients. Although recognized early as a possible respiratory therapy in hypoxemic failure, there was reluctance to use of HFNO due to presumed risk of transmission of the virus in the aerosols produced while using HFNO at the start of the pandemic [13]. Unfortunately, the use of HFNO is not in our database to further analyze this. The variation in the use of mechanical ventilation is seen in cohorts worldwide [14].

The length of ICU stay of patients was lower in Waves 2 and 3. Since patients in our cohort showed a stable disease severity and an increase in the number of comorbidities, the opposite, i.e. an increase of length of stay, would have been expected in Wave 2. In our view the decrease in length of ICU stay could have had several reasons. The first possibility is a change in the treatment for the most characteristic symptom of COVID-19 patients, hypoxia. As we showed, there was a decrease in use of mechanical ventilation. On the other hand, since invasive mechanical ventilation has inherent negative effects and may cause damage to lung, it could be that COVID-19 patients benefited from this change [15].

The second possibility is that because commencing mechanical ventilation requires the use of muscle relaxants and sedatives and use of the latter is known to lead to an increase in the length of stay [16]. Both mechanical ventilation and sedation are only applied when necessary and are lifesaving treatments. Yet another possibility would be that the ubiquitous use of dexamethasone led to a change in disease pattern, i.e. patients were further in their disease process when admitted to the ICU [17]. This is supported by the fact that patients stayed longer in the ward before being admitted to the ICU. Our results show that between Wave 1 and the Wave 3 the patients stayed on average almost a day longer on the ward. This may be explained by increased testing capacity over time leading to earlier diagnosis within the disease trajectory of individual patients affecting earlier hospital admission. Another possibility might have been that extra resources in the Wave 1 were more difficult to organize than in Waves 2 and 3. Not only bed utilization rates were higher but also extra personnel was not yet sufficiently trained during Wave 1, which might have affected quality of care [18].

The crude in-hospital mortality of COVID-19 ICU patients in our study remained at the same level of around 30% and declined in Wave 3 to below 25%. The fact that medication such as corticosteroids and tociluzimab had become standard of care may explain a part of this reduction. Second and more importantly, the vaccination programme commenced focusing on old and or fragile groups first. After adjustment for case-mix and other confounders this difference in mortality between the different periods was still apparent. Some studies show markedly different mortality levels, for instance the French cohort showed an ICU mortality of 50% [8]. In a Brazilian cohort, a striking different course of mortality over time was shown, in-hospital mortality decreased from a low 18.0% to an even lower 9.8% [9]. However, in a systematic review describing mainly the first wave and including 32 papers, the mean ICU mortality was comparable with our study, i.e., 30% [7].

A major strength of our study was that we were able to report on the first and second pandemic waves in the Netherlands covering all COVID-19 patients admitted to the ICU nationwide, serving a population of around 17.5 million people. We used an existing quality registry which for the COVID-19 pandemic was serving as one of the nation’s ways to track and model the pandemic in the Netherlands. Notably, transferred patients’ stays in multiple ICUs were combined, thus including each patient only once in our cohort, which is a major advantage over other cohorts that did not combine multiple stays at different ICUs. A limitation of our approach is that we did not have information on treatment protocols applied to the patients in our cohort. In the Netherlands, the use of dexamethasone and toziluzimab, as well as anticoagulant prophylaxis and other treatment, in these patients was directed by the scientific communities and institutions [19]. We also have no information on the use of non-invasive mechanical ventilation in relation to the use of invasive mechanical ventilation. We only have data on the use of mechanical ventilation as a whole. In addition, important reported comorbidities such as hypertension and hypercholesterolemia were not available in our data set [20]. Yet, another limitation is that we do not have data on the way COVID-19 was confirmed per patient. The fact is that in the second wave there was sufficient capacity to confirm the diagnosis with a PCR, whereas in the first wave, we had to rely more on radiologic confirmation. The change in confirmation method might have slightly affected the number of false positives and/ or negatives. However, we do not expect that this has influenced the observed mortality rate in COVID-19 patients in our cohort. Since COVID-19 which leads to an ICU admission is a clinical diagnosis, the confirmation is done as one needs to be as certain of the diagnosis as possible. We are confident that the vast majority of patients was correctly diagnosed with COVID-19 notwithstanding the confirmation method.

## Conclusions

In this study, the subsequent pandemic waves of ICU admissions due to SARS-CoV-2 infection in the Netherlands were compared. We found a shift to patients who were slightly older and had more comorbidities in the first two upsurges of Wave 2 and a decline in age and number of comorbidities in the final upsurge of Wave 2 (‘Wave 3’). In Waves 2 and 3 a favorable shift in treatment characteristics with shorter ICU stay, less relocations, and less mechanical ventilation was seen. This indicates more efficient care for COVID-19 patients admitted to the ICU. Moreover, the high ICU occupancy rate early in the pandemic does probably not explain the high mortality associated with COVID-19. The adjusted in-hospital mortality decreased in Wave 3.

## Supplementary Information

**Additional file 1:** **Figure S1.** Mean occupancy rate at the ICU (as fraction of the average number of patients in 2019) per week during the COVID-19 waves. **Table S1.** Patient characteristics. **Table S2.** Crude and adjusted logistic regression showing Odds ratios of hospital death during Wave 2, Wave 3, and the period in-between compared to Wave 1 (see Fig. 2). **Table S3.** Crude and adjusted Cox regression showing higher Hazard ratios of ICU discharge during Wave 2, Wave 3, and the period in-between compared to Wave 1 (see Fig. 3). A Hazard ratio of ICU discharge higher than 1.00 implies a comparatively high rate of discharge and, thus, a shorter length of stay at the ICU. Shown are an analysis with death as ICU discharge event and an analysis with death as censoring event.

## Data Availability

De-identified aggregated data used for the study will be made available upon reasonable request.

## Abbreviations

COVID-19: Corona Virus Disease 2019
ICU: Intensive Care Unit
NICE: The National Intensive Care Evaluation
OR: Odds Ratio
HR: Hazard Ratio
SARS-CoV-2: Severe Acute Respiratory Syndrome Corona Virus-2
HFNO: High flow nasal oxygen
RT-PCR: Reverse transcription polymerase chain reaction
CO-RADS: COVID-19 Reporting and Data System
BMI: Body Mass Index
APACHE-IV: Acute Physiology and Chronic Health Evaluation IV
