# Supplementary material for: Characteristics and outcome of COVID-19 patients admitted to the ICU: a nationwide cohort study on the comparison between the first and the consecutive upsurges of the second wave of the COVID-19 pandemic in the Netherlands
Source: Ann Intensive Care. 2022 Jan 13;12:5. doi: 10.1186/s13613-021-00978-3 (PMC8755895; doi:10.1186/s13613-021-00978-3)
Supplement: Supplementary file 1 — Additional file 1: Figure S1. Mean occupancy rate at the ICU (as fraction of the average number of patients in 2019) per week during the COVID-19 waves. Table S1. Patient characteristics. Table S2. Crude and adjusted logistic regression showing Odds ratios of hospital death during Wave 2, Wave 3, and the period in-between compared to Wave 1 (see Fig. 2). Table S3. Crude and adjusted Cox regression showing higher Hazard ratios of ICU discharge during Wave 2, Wave 3, and the period in-between compared to Wave 1 (see Fig. 3). A Hazard ratio of ICU discharge higher than 1.00 implies a comparatively high rate of discharge and, thus, a shorter length of stay at the ICU. Shown are an analysis with death as ICU discharge event and an analysis with death as censoring event. [file 13613_2021_978_MOESM1_ESM.docx]

Supplement Figure 1. Mean occupancy rate at the ICU (as fraction of the average number of patients in 2019) per week during the Covid-19 waves.


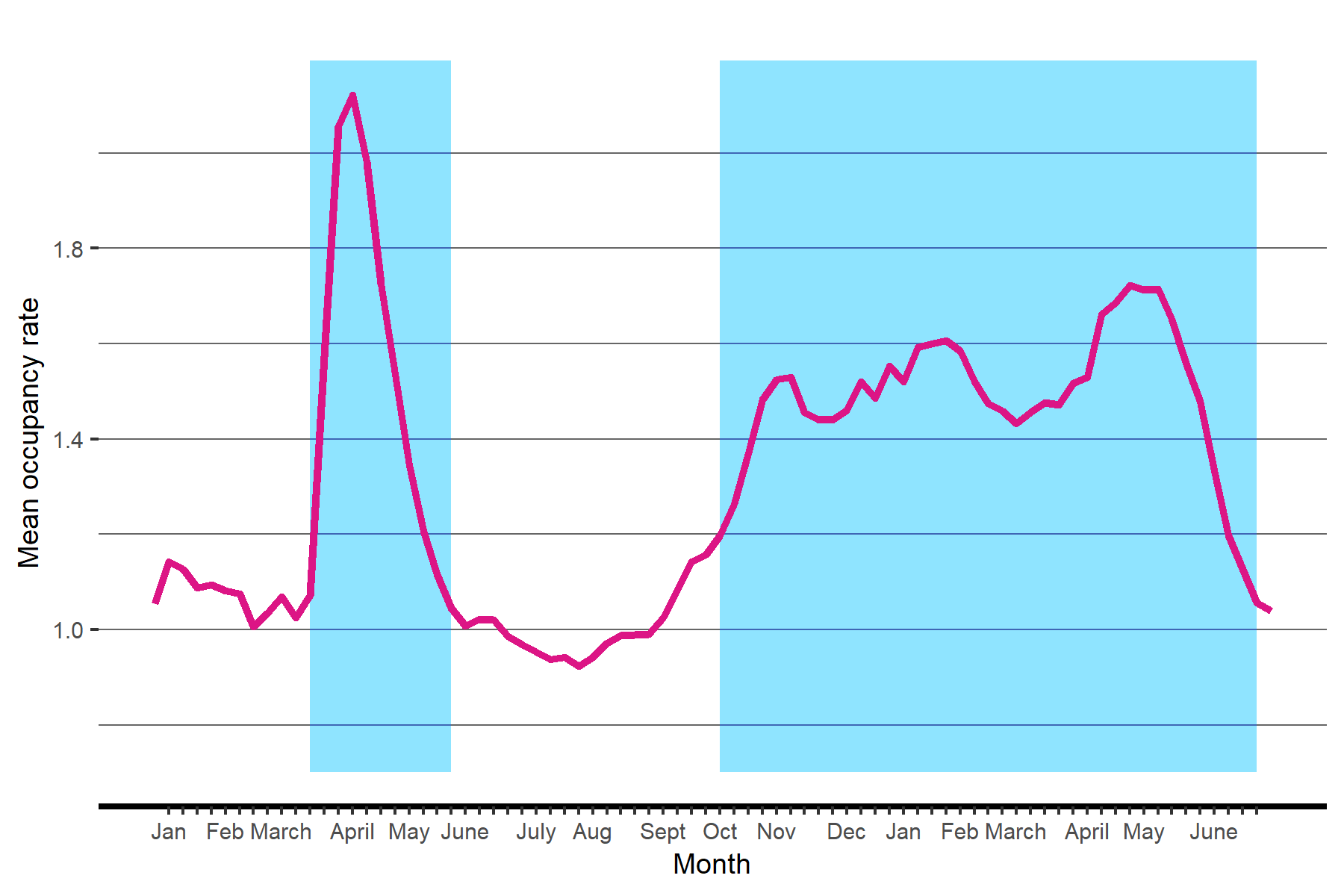


Supplement Table 1 Patient characteristics

| Characteristic | Total | Wave 1 | In-between | Wave 2 | Wave 3 | P value  Wave 2 vs Wave 1 | P value  Wave 3 vs Wave 1 |
| --- | --- | --- | --- | --- | --- | --- | --- |
| Age N (%) <40 years | 526 (4.4) | 104 (3.8) | 26 (5.4) | 151 (3.6) | 245 (5.3) | <0.001 | <0.001 |
| 40-45 years | 312 (2.6) | 52 (1.9) | 21 (4.4) | 73 (1.7) | 166 (3.6) |  |  |
| 45-50 years | 628 (5.2) | 153 (5.6) | 39 (8.1) | 176 (4.2) | 260 (5.6) |  |  |
| 50-55 years | 1133 (9.4) | 243 (8.9) | 52 (10.8) | 332 (7.9) | 506 (11) |  |  |
| 55-60 years | 1573 (13.1) | 358 (13.1) | 51 (10.6) | 497 (11.8) | 667 (14.5) |  |  |
| 60-65 years | 1843 (15.3) | 426 (15.6) | 61 (12.7) | 678 (16.1) | 678 (14.7) |  |  |
| 65-70 years | 2111 (17.5) | 486 (17.8) | 76 (15.8) | 730 (17.3) | 819 (17.8) |  |  |
| 70-75 years | 2247 (18.7) | 542 (19.8) | 78 (16.2) | 859 (20.4) | 768 (16.7) |  |  |
| 75-80 years | 1281 (10.6) | 300 (11) | 51 (10.6) | 523 (12.4) | 407 (8.8) |  |  |
| 80-85 years | 333 (2.8) | 59 (2.2) | 22 (4.6) | 174 (4.1) | 78 (1.7) |  |  |
| >85 years | 43 (0.4) | 10 (0.4) | 3 (0.6) | 22 (0.5) | 8 (0.2) |  |  |
| Comorbidities N (%) |  |  |  |  |  |  |  |
| Innumological insufficieny | 1103 (9.2) | 207 (7.6) | 40 (8.3) | 450 (10.7) | 406 (8.8) | <0.001 | 0.0682 |
| Renal insufficiency | 497 (4.1) | 73 (2.7) | 19 (4) | 248 (5.9) | 157 (3.4) | <0.001 | 0.091 |
| Respiratory insufficiency | 1530 (12.7) | 305 (11.2) | 48 (10) | 614 (14.6) | 563 (12.2) | <0.001 | 0.1805 |
| Malignancy | 307 (2.6) | 66 (2.4) | 12 (2.5) | 143 (3.4) | 86 (1.9) | 0.0239 | 0.1329 |
| Cardiovascular diseases | 189 (1.6) | 33 (1.2) | 7 (1.5) | 84 (2) | 65 (1.4) | 0.0168 | 0.526 |
| Cirrhosis | 49 (0.4) | 3 (0.1) | 2 (0.4) | 22 (0.5) | 22 (0.5) | 0.0094 | 0.016 |
| APACHE-IV mortality probability (in quintiles) N (%) |  |  |  |  |  |  |  |
| <12.2 | 2401 (20.0) | 594 (21.7) | 137 (28.5) | 710 (16.8) | 960 (20.9) | <0.001 | <0.001 |
| 12.2 – 18.8 | 2400 (20.0) | 539 (19.7) | 89 (18.5) | 800 (19) | 972 (21.1) |  |  |
| 18.8-26.3 | 2400 (20.0) | 510 (18.7) | 80 (16.7) | 824 (19.5) | 986 (21.4) |  |  |
| 26.3-37.9 | 2400 (20.0) | 546 (20.0) | 76 (15.8) | 872 (20.7) | 906 (19.7) |  |  |
| 37.9-98.9 | 2401 (20.0) | 542 (19.8) | 96 (20.0) | 998 (23.7) | 765 (16.6) |  |  |
| Unknown | 28 (0.20) | 2 (0.1) | 2 (0.4) | 11 (0.3) | 13 (0.3) |  |  |
| PaO2 (mmHg, in quintiles) N(%) |  |  |  |  |  |  |  |
| <59 | 1994 (16.6) | 278 (10.2) | 74 (15.4) | 772 (18.3) | 870 (18.9) | <0.001 | <0.001 |
| 59-68 | 2318 (19.3) | 400 (14.6) | 86 (17.9) | 826 (19.6) | 1006 (21.9) |  |  |
| 68-76 | 1967 (16.4) | 439 (16.1) | 77 (16.0) | 682 (16.2) | 769 (16.7) |  |  |
| 76-91 | 2313 (19.2) | 623 (22.8) | 93 (19.4) | 759 (18) | 838 (18.2) |  |  |
| >91 | 2201 (18.3) | 699 (25.6) | 88 (18.3) | 708 (16.8) | 706 (15.3) |  |  |
| Unknown | 1237 (10.3) | 294 (10.8) | 62 (12.9) | 468 (11.1) | 413 (9.0) |  |  |

Wave 1, February 2020 – May 24, 2020

Period in-between, May 25, 2020 – October 4, 2020

Wave 2: first and second upsurge of Wave 2, October 5, 2020 – January 31, 2021

Wave 3: final upsurge of Wave 2, February 1 – June 30, 2021

Supplement Table 2. Crude and adjusted logistic regression showing Odds ratios of hospital death during Wave 2, Wave 3, and the period in-between compared to Wave 1 (see Figure 2).

|  | Wave 1 (reference) | In-between | Wave 2 | Wave 3 |  |
| --- | --- | --- | --- | --- | --- |
|  |  | OR (95% CI) | OR (95% CI) | OR (95% CI) | Wald X2, df, P value |
| Crude | 1.00 | 0.84 (0.67-1.05) | 1.11 (1.0 – 1.24) | 0.73 (0.65-0.81) | 79.377, 3, <0.001 |
| Adjusted for age, gender, BMI, and APACHE-IV risk | 1.00 | 0.89 (0.69-1.14) | 0.98 (0.87-1.1) | 0.79 (0.7-0.89) | 21.217, 3, <0.001 |
| Adjusted for age, gender, BMI, APACHE-IV risk and ICU occupancy rate | 1.00 | 0.85 (0.65-1.1) | 0.98 (0.87-1.11) | 0.80 (0.71-0.9) | 20.281, 3, <0.001 |

Wave 1, February 2020 – May 24, 2020

Period in-between, May 25, 2020 – October 4, 2020

Wave 2: first and second upsurge of Wave 2, October 5, 2020 – January 31, 2021

Wave 3: final upsurge of Wave 2, February 1 – June 30, 2021

Supplement Table 3. Crude and adjusted Cox regression showing higher Hazard ratios of ICU discharge during Wave 2, Wave 3, and the period in-between compared to Wave 1 (see Figure 3). A Hazard ratio of ICU discharge higher than 1.00 implies a comparatively high rate of discharge and, thus, a shorter length of stay at the ICU. Shown are an analysis with death as ICU discharge event and an analysis with death as censoring event.

|  | Wave 1 (reference) | In-between | Wave 2 | Wave 3 |  |
| --- | --- | --- | --- | --- | --- |
|  |  | HR (95%-CI) | HR (95%-CI) | HR (95%-CI) | Wald X2, df, P value |
| Death as ICU discharge event |  |  |  |  |  |
| Crude | 1.00 | 1.27 (1.15-1.4) | 1.20 (1.14-1.26) | 1.30 (1.24-1.37) | 119.687, 3, <0.001 |
| Adjusted for age, gender, BMI, and APACHE-IV risk | 1.00 | 1.23 (1.11-1.36) | 1.26 (1.2-1.32) | 1.35 (1.28-1.41) | 148.518, 3, <0.001 |
| Adjusted for age, gender, BMI, APACHE-IV risk and ICU occupancy rate | 1.00 | 1.17 (1.05-1.30) | 1.22 (1.16-1.28) | 1.32 (1.26-1.39) | 127.101, 3, <0.001 |
|  |  |  |  |  |  |
| Death as censoring event |  |  |  |  |  |
| Crude | 1.00 | 1.34 (1.19-1.5) | 1.17 (1.11-1.24) | 1.41 (1.33-1.49) | 154.021, 3, <0.001 |
| Adjusted for age, gender, BMI, and APACHE-IV risk | 1.00 | 1.33 (1.19-1.49) | 1.31 (1.23-1.38) | 1.45 (1.37-1.54) | 172.034, 3, <0.001 |
| Adjusted for age, gender, BMI, APACHE-IV risk and ICU occupancy rate | 1.00 | 1.28 (1.13-1.44) | 1.26 (1.19-1.34) | 1.42 (1.34-1.5) | 145.646, 3, <0.001 |

Wave 1, February 2020 – May 24, 2020

Period in-between, May 25, 2020 – October 4, 2020

Wave 2: first and second upsurge of Wave 2, October 5, 2020 – January 31, 2021

Wave 3: final upsurge of Wave 2, February 1 – June 30, 2021
